# Risk Factors of Stunting and Wasting among Children Aged 6–59 Months in Household Food Insecurity of Jima Geneti District, Western Oromia, Ethiopia: An Observational Study

**DOI:** 10.1155/2022/3981417

**Published:** 2022-01-13

**Authors:** Tamiru Yazew

**Affiliations:** Department of Food and Nutritional Sciences, Shambu Campus, Wollega University, Shambu, Ethiopia

## Abstract

Undernutrition is the most difficult and widespread public health concern in low-income nations including Ethiopia. Therefore, this study aimed to investigate the associated risk factors of stunting and wasting among children aged 6–59 months in Jima Geneti district, Western Oromia, Ethiopia. A community-based cross-sectional study was conducted on 500 children from December 1 to 28, 2020. A multiple-stage sampling method was performed to select children from each kebele. Anthropometric measurements were taken, and the nutritional status was generated using WHO Anthro v. 3.2.1. Data analysis was performed using the SPSS version 20.0. Bivariate and multivariate logistic regression analyses were carried out to identify the associated risk factors of stunting and wasting among children in the study area. Statistical significance was set at *p* < 0.05. The study results showed that the prevalence of stunting and wasting among children was 27% and 11.8%, respectively. The findings of this study also revealed that the prevalence of household food insecurity and poor dietary diets was 19.6% and 52.2%, respectively. Low wealth status (AOR = 2.5; 95% CI: 1.1, 5.55) and poor dietary diets (AOR = 4.7; 95% CI: 2.5, 8.83) were associated risk factors for stunting. However, child meal frequency (AOR = 3.9; 95% CI: 1.23, 12.6), and children who did feed leftover food (AOR = 2.75; 95% CI: 1.02, 7.44) were associated risk factors for wasting. Poor dietary diets (AOR = 2.65; 95% CI: 1.06, 6.66) were also associated risk factors for wasting. The findings of this study concluded that the prevalence of stunting and wasting was high in the study area. Therefore, addressing family-level risk factors which are major drivers of children's nutritional status is crucial to ensure the nutritional status of children.

## 1. Introduction

Globally, the prevalence of stunting and wasting among children under five years of age was 29.1% and 6.3%, respectively [1]. Annually, one-third of all deaths in children are due to acute and chronic undernutrition [2]. A substantially higher estimated prevalence of undernutrition was found in Africa, Southern Asia, and Southeast Asia than global average estimates [1]. In Ethiopia, it has been reported that the prevalence of stunting and wasting was 37% and 7%, respectively [3]. Undernutrition causes morbidity and mortality among children, and food insecurity is more prevalent [4]. Its high rates may also pose a significant obstacle to achieving better child health and nutritional outcomes [5].

Even though the government of the country was planning different interventions and programs to combat undernutrition and food insecurity, Ethiopia is still highly vulnerable to poverty, household food insecurity, and child undernutrition [6]. These problems are very prevalent, particularly in poor communities whose livelihoods depend on the backward farming system [7].

Several risk factors associated with stunting among children have also been identified, including a low wealth index [8–10]. Another associated risk factor of stunting was poorly diversified diets [11–14]. Moreover, studies conducted in Ethiopia reported that the sex of the child, age of the child, and immunization status were the major associated risk factors of stunting [15–17]. Poverty level, education, occupation, household food insecurity, and dietary diets were also risk factors associated with stunting [18].

Similarly, lack of a balanced diet, housing quality, and water quality were other associated risk factors for wasting [19]. Family size, diarrhea [15], and prelacteal feeding [20] have also been reported as the major risk factors associated with wasting. Other associated risk factors for wasting were also household poverty level, educational status, occupational status, household food insecurity, and dietary diets [18]. In addition, birth order and no antenatal care visits have also been identified as risk factors associated with wasting [9, 21, 22].

The Oromia region produces adequate food in the country. However, it is reported to have a high prevalence rate of undernutrition compared to the less productive regions of Ethiopia [21]. In the Jima Geneti district, most of the kebeles suffer from food insecurity due to heavy rainfall and less agricultural production. However, no study has assessed the associated risk factors with stunting and wasting among children under five years of age in the study area. Therefore, this study was initiated to assess associated risk factors of stunting and wasting among children aged 6–59 months in Jima Geneti district, Western Oromia, Ethiopia.

## 2. Materials and Methods

### 2.1. Study Setting, Design, and Period

This cross-sectional study was carried out from December 1 to 28, 2020 in the Jima Geneti district, Western Oromia, Ethiopia. Jima district was purposively selected as the study site because many rural communities in the study areas were dependent on food aid and support for over ten years [22]. The 2007 national census reported a total population of 64,158, of whom 31,756 were men and 32,402 were women; 6,966 (10.86%) of its population were urban dwellers. A community-based cross-sectional study design was performed for this study. This procedure was reproduced from a previous study conducted in Jima Geneti district, Ethiopia [23]. All children aged 6–59 months found in the study area, Jima district was the target for the study, whereas the study population consisted of a sample of all households with 6–59 months old children who were residing in randomly selected kebeles. Mothers with children aged 6–59 months who had resided in the Jima Geneti district for at least six months were included in this study. Children aged 6–59 months who had deformities or were chronically sick, as well as mothers who were deaf, were excluded from this study.

### 2.2. Sample Size Determination

The sample size of the study was calculated using a formula for a single population proportion by considering the following assumptions: 95% CI, *P* = expected prevalence of stunting in the Oromia region, which was 27% [21]. Therefore, the total sample size required for this study was 500 children by considering a 10% nonresponse rate and a design effect of 1.5.

### 2.3. Sampling Procedures

The Jima Geneti district was purposively selected for this study site because there has been no study conducted prior study regarding the assessment of risk factors of stunting and wasting among children aged 6–59 months. The study area suffered from heavy rainfall and reduced agricultural production. A multistage sampling method was used to draw the samples for the study. From the 14 kebeles, five kebeles Gudetu Jima, Hunde Gudina, Gudetu Geneti, Lalisa Biya, and Kelela Didimtu were selected using a simple random sampling technique. The children in each kebele were also selected using population proportion to size allocation based on the available data at the Horo Guduru Wollega zonal health office. Finally, from each kebele, the eligible children required for the study were selected randomly.

### 2.4. Data Collection

#### 2.4.1. Data Collection Procedures

Socioeconomic and demographic data were collected using a structured questionnaire adapted from relevant studies. The questionnaire was first developed in English and translated into a survey (Afan Oromo) language. Then it was translated back to English by different language experts to check for consistency. To manage the quality of the data, a pretest was performed on 5% of households that were not included in the actual samples of the study. To improve the quality of the data, three days of training were provided to all data collectors and supervisors. Questionnaires were also pretested on 5% of households that were not included in the actual samples of the study. At the end of each day, the completeness of the questionnaires was checked by the principal investigator.

#### 2.4.2. Sociodemographic and Economic Data

The sociodemographic (sex, and age of child, parents' educational and occupational status, marital status) and economic factors (wealth index, ownership of land or cattle) were collected from mothers/caregivers.

#### 2.4.3. Dietary Diversity Data

The dietary diversity scores (DDS) and 24-hour recall method were conducted regarding the child's diet intake in the 24 hours preceding the survey. Mothers/caregivers were requested to list all the foods consumed by the child in the 24 hours preceding the interview. Seven food groups were used for eligible children. Considering four food groups as the minimum acceptable dietary diversity, a child with a diet diversity score of <4, was classified as having poor dietary diversity and high if a child's dietary diversity score was [24, 25].

#### 2.4.4. Household Food Insecurity Data

Household food insecurity (HFI) was measured using the Household Food Insecurity Access Scale (HFIA), which has nine questions and is related to the households' experience of food insecurity in the 12 months preceding the survey [26]. Then, the HFIA prevalence indicators were categorized households into four (4) levels of HFI: food-secure, mild, moderately, and severely food insecure. For this study, only two levels of the Household Food Insecurity Access Scale (food secure and insecure) were used because the sample size for this study was small.

#### 2.4.5. Anthropometric Data

Anthropometric measurements were taken using the procedures of the World Health Organization guidelines [27]. The weight of the child was measured using an electronic digital weight scale. Wooden height scales were used to measure height. Anthropometric tools were calibrated before each day of data collection. The age of the child was calculated in months from the birth date to the day of data collection using a local event and childbirth certificate.

### 2.5. Variables of the Study

In this study, stunting and wasting of children were used as dependent variables, while household sociodemographic and economic characteristics, food insecurity, child feeding practices, and dietary diversity were independent variables.

### 2.6. Ethical Consideration

Ethical clearance was obtained from the Institutional Review Board (IRB) of the Wollega University, College of Health Sciences. A formal letter of permission was written to the Regional Health Office and each selected kebele administration. After explaining the purpose of the study, verbal consent was obtained from each study participant. Participants were informed that participation was voluntary. Personal identifiers were not included in the questionnaires to ensure participants' confidentiality. Nutrition and health education was given by the data collectors for households and those mothers who had malnourished children were advised to go to health facilities for further treatment.

### 2.7. Statistical Analysis

The data obtained were analyzed using the SPSS version 20.0. The nutritional status of children was generated using the WHO Anthro program, version 3.2.1. Children with a height for age and weight for height *Z* score less than -2 standard deviations or below the standard median were considered as stunted and wasted, respectively. Logistic regression was conducted to identify associated risk factors with stunting and wasting. A *p* value <0.05 was declared as statistically significant. The degree of association between dependent and independent variables was reported using an adjusted odds ratio (AOR) with 95% CI.

## 3. Results

### 3.1. Sociodemographic and Economic Characteristics

Of the total participants, 472 (94.4%) were married. About 406 (81.2%) mothers had no formal education. More than half of the participants (62.6%) had a family size of >5. In addition, 296 (59.2%), 113 (22.6%), and 91 (18.2%) households were categorized as low-, medium-, and high-income households, respectively (see Table 1).

### 3.2. Child Dietary Diversity Scores

**Figure 1:**
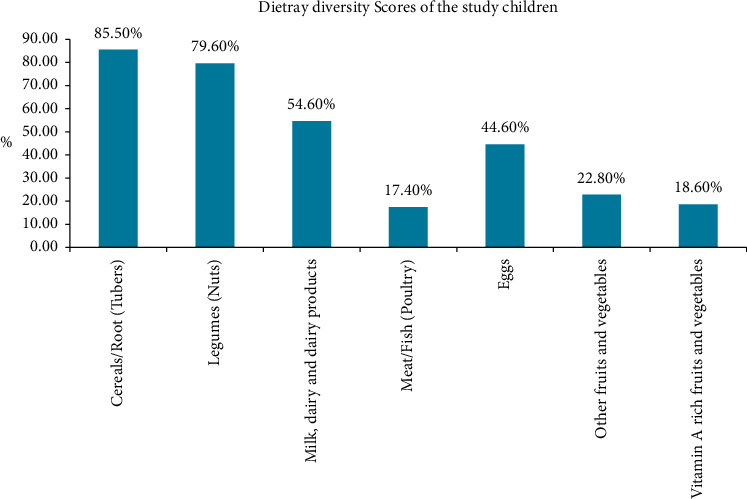
Dietary diversity scores of children.

Figure 1 illustrates that approximately about 428 (85.5%) and 398 (79.6%) children consumed cereal and legume-based foods, respectively. In addition, about 87 (17.4%) children did not receive meat/fish/poultry for consumption. Regarding the dietary diversity score, 239 (47.8%) and 261 (52.2%) children had high (≥4) and poor (≤3) dietary diversity scores, respectively.

### 3.3. Household Food Security Status

Table 2 indicates that about 143 (28.6%) households were unable to eat the kinds of foods they preferred. These findings also report that about 313 (62.6%) and 123 (24.6%) households ate a smaller meal than they felt was needed and ate fewer meals per day, respectively. In addition, about 106 (21.2%) households slept at night hungry. About 402 (80.4%) and 98 (19.6%) households were classified as household food security and insecurity, respectively. Table 2 is reproduced from a previous study done in Ethiopia [23].

### 3.4. Prevalence of Stunting and Wasting

**Figure 2:**
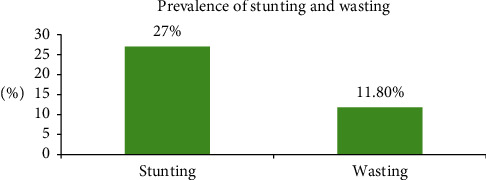
Prevalence of stunting and wasting.

Out of 500 children, the prevalence of stunting and wasting among children aged 6–59 months was 27% and 11.8%, respectively (see Figure 2).

### 3.5. Risk Factors of Stunting and Wasting

Table 3 shows that in the multivariate logistic regression analysis, low wealth status (AOR = 2.5; 95% CI: 1.1, 5.55) and poor dietary diets (AOR = 4.7; 95% CI: 2.5, 8.83) were risk factors for stunting, whereas child meal frequency (AOR = 3.9; 95% CI: 1.23, 12.6), children who did feed leftover food (AOR = 2.75; 95% CI: 1.02, 7.44), and poor dietary diets (AOR = 2.65; 95% CI: 1.06, 6.66) were risk factors for wasting (see Table 4).

## 4. Discussion

Despite various interventions and policies like National Nutrition and Nutrition-Sensitive to Agriculture being established to overcome risk factors of undernutrition in Ethiopia, the prevalence of stunting and wasting among children in Ethiopia and the study area was still high. The findings of this study illustrated that the prevalence of stunting and wasting in food-insecure households was 27% and 11.8%, respectively.

This finding was consistent with a study conducted in the Tigray region of Ethiopia, which showed that the prevalence of stunting and wasting among children in household food insecurity was 52.1% and 12.6%, respectively [28]. It also agreed with a study conducted in west Oromia, which showed that the prevalence of stunting and wasting among children in food-insecure households was 41.8% and 14.9%, respectively [29]. A similar report in South Ethiopia showed that the prevalence of stunting and wasting among children in household food insecurity was 45.6% and 14.6%, respectively [30]. Moreover, this study result is agreed with a study conducted in Malaysia, which explored that the prevalence of childhood stunting and wasting in household food insecurity was 54.7% and 27%, respectively [31]. However, the prevalence of stunting and wasting among children in the current was lower than the above study findings. This variation might be due to sample size, data collection period, and inequalities in socioeconomic and demographic conditions of the current study.

According to the finding of this study, children in low-income households have an increased likelihood of being stunted than those who were in the high income. This finding was consistent with studies done in Bangladesh [32, 33] and Ethiopia [34, 35].

The finding of this study also reported that children who had an undiversified diet were more likely to be stunted than those who had high dietary diversity scores. The study result was supported by studies conducted in Ethiopia [14, 18, 36]. This might be due to poor dietary diversity scores may not providing all the essential nutrients required for the child's growth and mental development.

The multivariate analysis of this study also revealed that children who did get poor dietary diets were more likely to be wasted than those who had high diets. This finding was in line with studies conducted in Ethiopia [18, 37]. Moreover, the present study found that those children who had meal frequency/day ≤3 were more likely to be wasted than that of the reference group (AOR = 1.0). This study result was consistent with those of a study result conducted in west Gojjam [37]. Furthermore, children who did feed leftover food were more likely to be wasted than those who did not. Similar results were reported in Ethiopia [38, 39]. This might be because leftover foods have unhygienic and are contaminated with lots of microorganisms that can cause the loss of the weight of the children.

## 5. Conclusions

It was observed that the prevalence of stunting and wasting among children aged 6–59 months in household food insecurity in the Jima Geneti district was high. The study findings concluded that low wealth status and poor dietary diets were associated risk factors of stunting. Child meal frequency, the child who did feed leftover foods, and poor dietary diets were also associated risk factors of wasting.

### 5.1. Limitation of the Study

This survey was conducted in a single period, and this may not explain the true children's dietary diets and their nutritional status. The households being grouped as food secure and insecure based on productivity safety net program criteria may not also indicate the current food security status of the surveyed households; as many households classified as food secure were being out of the program for more than ten years, this may increase bias in the research. Anthropometric measurement errors and recall biases in dietary diversity and food insecurity could exist.

### 5.2. Recommendations

Based on the findings of this study, the following points are recommended:

- Providing health and nutrition education through behavioral change communication is crucial to tackling associated risk factors of malnutrition
- Nutrition and health information should be given by health extension workers for mothers as poor child feeding practices were a major factor among children in the study area
- Encouraging households to use home-gardening systems and rearing small animals is very important to diversify child's food and ensure their household economic status

## Figures and Tables

**Table 1:** Sociodemographic and economic characteristics of the study participants.

Variables	Frequency	Percent
Age of the child (in months)		
6–11	130	26.0
12–24	156	31.2
25–47	101	20.2
48–59	113	22.6
Sex of the child		
Male	203	40.6
Female	297	59.4
Birth order of the child		
First	86	17.2
Second	99	19.8
Third and above	315	63
Maternal age (in years)		
< 25 years	124	24.6
25–36 years	218	43.6
≥36 years	158	31.6
Religion		
Orthodox	82	16.4
Muslim	117	23.4
Protestant and others	301	60.2
Marital status of the mother		
Single	8	1.6
Married	472	94.4
Divorced and others	20	4
Education status of the mother		
Attend formal education	94	18.8
Not attend formal education	406	81.2
Occupational status of the mother		
Housewife	369	73.8
Farmer	12	2.4
Gov't employment	24	4.8
Merchant	57	11.4
Daily labor	38	7.6
Education status of the mother		
Attend formal education	123	24.6
Not attend formal education	377	75.4
Occupational status of the father		
Farmer	371	74.2
Gov't employment	56	11.2
Merchant	29	5.8
Daily labor	44	8.8
Household family size		
<5	187	37.4
≥5	313	62.6
Household wealth index		
Low	296	59.2
Medium	113	22.6
High	91	18.2

**Table 2:** Household food security status of the participants.

Variables	Frequency	Percent
Worried about not having enough food		
Yes	86	17.2
No	414	82.8
Not able to eat the kinds of foods he/she preferred		
Yes	143	28.6
No	357	71.4
Ate just a few kinds of food day after day		
Yes	119	23.8
No	381	76.2
Ate food that he/she preferred not to eat		
Yes	247	49.4
No	253	50.6
Ate a smaller meal than he/she felt was needed		
Yes	313	62.6
No	187	37.4
Ate fewer meals in a day		
Yes	123	24.6
No	377	75.4
No food at all		
Yes	43	8.6
No	457	91.4
Went to sleep at night hungry		
Yes	106	21.2
No	394	78.8
Household food security status		
Food secure	402	80.4
Food insecure	98	19.6

Source: [23].

**Table 3:** Associated risk factors of stunting.

Variables	No. (%)	COR (CI)	AOR (CI)
Wealth status			
High	98 (19.6)	1.0	1.0
Medium	176 (35.2)	1.7 (0.92, 3.11)^*∗*^	1.4 (0.71, 2.76)
Low	226 (45.2)	3.1 (1.52, 6.32)^*∗*^	2.5 (1.1, 5.55)^*∗∗*^
Child meal frequency/day			
>=4	112 (22.4)	1.0	1.0
<=3	388 (77.6)	2.63 (1.65, 4.2)^*∗*^	0.75 (0.31, 1.8)
Child has breakfast			
Yes	135 (27.0)	1.0	1.0
No	365 (73.0)	3.46 (2.14, 5.6)^*∗*^	2.04 (0.46, 3.8)
The child has a midmorning snack			
Yes	259 (51.8)	1.0	1.0
No	241 (48.2)	1.88 (1.923, 3.2)^*∗*^	0.92 (0.46, 1.85)
The child has afternoon snacks			
Yes	234 (46.8)	1.0	1.0
No	266 (74.2)	2.48 (1.6, 3.92)^*∗*^	1.46 (0.72, 2.96)
The child has bedtime snacks			
Yes	137 (27.4)	1.0	1.0
No	363 (72.6)	1.89 (2.9, 3.86)^*∗*^	1.13 (0.46, 2.8)
When does the child eat his food?			
Upon the child demands	320 (64.0)	1.0	1.0
When convenient for the mother	180 (36.0)	1.72 (1.02, 2.9)^*∗*^	0.73 (0.37, 1.44)
Did you restrict the child during his/her meal?			
No	246 (49.2)	1.0	1.0
Yes	254 (50.8)	2.44 (1.52, 3.9)^*∗*^	1.16 (0.65, 2.08)
Did you pressure the child to eat his food?			
No	251 (50.2)	1.0	1.0
Yes	249 (49.8)	1.82 (1.16, 2.9)^*∗*^	0.99 (0.55, 1.77)
Was food leftover in your home in the past 24 hrs day?			
No	241 (48.2)	1.0	1.0
Yes	259 (51.8)	1.44 (0.91, 2.3)^*∗*^	1.6 (0.92, 2.8)
What do you do when food is leftover at home?			
Give to the animal	268 (53.6)	1.0	
Give to the child	232 (46.4)	1.06 (0.57, 1.96)	
Does your child eat all types of food?			
Yes	298 (59.6)	1.0	1.0
No	202 (40.4)	2.17 (1.35, 3.5)^*∗*^	1.6 (0.92, 2.8)
Dietary diversity scores			
High dietary diversity	178 (35.6)	1.0	1.0
Low dietary diversity	322 (64.4)	5.69 (3.3, 9.94)^*∗*^	4.7 (2.5, 8.83)^*∗∗*^

COR: crude odds ratio; AOR: adjusted odds ratio; ^*∗*^ statistically significant differences were observed at *p* < 0.05; 1 = references.

**Table 4:** Associated risk factors of wasting.

Variables	No. (%)	COR (CI)	AOR (CI)
Wealth status			
High	103 (20.6)	1.0	1.0
Medium	184 (36.8)	1.86 (1.52, 4.1)^*∗*^	1.8 (0.655, 4.95)
Low	213 (42.6)	4.25 (1.76, 10.25)^*∗*^	2.84 (0.91, 8.9)
Child meal frequency/day			
>=4	179 (35.8)	1.0	1.0
<=3	321 (64.2)	3.1 (1.66, 5.79)^*∗*^	3.9 (1.23, 12.6)^*∗∗*^
Child has breakfast			
Yes	245 (49.0)	1.0	1.0
No	255 (51.0)	2.8 (1.5, 5.2)^*∗*^	1.73 (0.773, 3.87)
The child has a midmorning snack			
Yes	223 (44.6)	1.0	1.0
No	277 (55.4)	1.22 (0.65, 2.28)	0.52 (0.22, 1.245)
The child has afternoon snacks			
Yes	367 (73.4)	1.0	1.0
No	133 (26.6)	2.3 (0.24, 4.25)	1.16 (0.522, 2.57)
The child has a bedtime snack			
Yes	165 (33.0)	1.0	1.0
No	335 (67.0)	1.45 (0.688, 3.2)	0.885 (0.395, 1.98)
When does the child eat his food?			
When convenient for the mother	269 (53.8)	1.0	
Upon the child demands	231 (46.2)	1.77 (0.96, 3.277)	—
Did you restrict the child during his/her meal?			
No	179 (35.8)	1.0	1.0
Yes	321 (64.2)	1.34 (2.73, 2.45)^*∗*^	0.69 (.39, 1.46)
Did you pressure the child to eat his food?			
No	293 (58.6)	1.0	1.0
Yes	207(41.4)	1.87 (1.01, 3.43)^*∗*^	0.71 (0.3,1.64)
Was food leftover in your home in the past 24 hrs day?			
No	242 (48.4)	1.0	
Yes	258 (51.6)	2.88 (1.486, 5.6)	—
What do you do when food is leftover at home?			
Give to the animal	184 (36.8)	1.0	1.0
Give to the child later	316 (63.2)	2.48 (1.0, 6.12)^*∗*^	2.75 (1.02, 7.44)^*∗∗*^
Does your child eat all types of food?			
Yes	103 (20.6)	1.0	1.0
No	397 (79.4)	1.987 (1.075, 3.67)^*∗*^	0.757 (0.32, 1.8)
Dietary diversity scores			
High dietary diversity	136 (27.2)	1.0	1.0
Low dietary diversity	364 (71.8)	3.3 (1.64, 6.55)^*∗*^	2.65 (1.06, 6.66)^*∗∗*^

COR: crude odds ratio; AOR: adjusted odds ratio; ^*∗*^ statistically significant differences were observed at *p* < 0.05; 1 = references.

## Data Availability

All data underlying the study results are available from the corresponding author upon reseanable request.
